# Silica immobilization of *Geobacter sulfurreducens* for constructing ready‐to‐use artificial bioelectrodes

**DOI:** 10.1111/1751-7915.12561

**Published:** 2017-04-11

**Authors:** Marta Estevez‐Canales, David Pinto, Thibaud Coradin, Christel Laberty‐Robert, Abraham Esteve‐Núñez

**Affiliations:** ^1^ Department of Chemical Engineering University of Alcalá Alcalá de Henares Madrid Spain; ^2^ Sorbonne Universités UPMC Univ. Paris 06 CNRS Collège de France Laboratoire de Chimie de la Matière Condensée de Paris (LCMCP) 4 place Jussieu F‐75005 Paris France; ^3^ IMDEA Water Institute Alcalá de Henares Spain

## Abstract

Microbial electrochemical technologies (METs) rely on the control of interactions between microorganisms and electronic devices, enabling to transform chemical energy into electricity. We report a new approach to construct ready‐to‐use artificial bioelectrodes by immobilizing *Geobacter sulfurreducens* cells in composite materials associating silica gel and carbon felt fibres. Viability test confirmed that the majority of bacteria (ca. 70 ± 5%) survived the encapsulation process in silica and that cell density did not increase in 96 h. The double entrapment within the silica–carbon composite prevented bacterial release from the electrode but allowed a suitable mass transport (ca. 5 min after electron donor pulse), making the electrochemical characterization of the system possible. The artificial bioelectrodes were evaluated in three‐electrode reactors and the maximum current displayed was ca. 220 and 150 μA cm^−3^ using acetate and lactate as electron donors respectively. Cyclic voltammetry of acetate‐fed bioelectrodes revealed a sigmoidal catalytic oxidation wave, typical of more advanced‐stage biofilms. The presence of *G. sulfurreducens* within composites was ascertained by SEM analysis, suggesting that only part of the bacterial population was in direct contact with the carbon fibres. Preliminary analyses of the transcriptomic response of immobilized *G. sulfurreducens* enlightened that encapsulation mainly induces an osmotic stress to the cells. Therefore, ready‐to‐use artificial bioelectrodes represent a versatile time‐ and cost‐saving strategy for microbial electrochemical systems.

## Introduction

The advent of microbial fuel cells (MFC) marked the beginning of a new research area, between biology and electrochemistry (Schröder *et al*., 2015). These electrochemical devices enable the transformation of chemical energy into electricity by means of electroactive microorganisms. They are able to oxidize organic compounds (electron donors) to reduce an electrode as an extracellular electron acceptor (Kim *et al*., 2002; Bond and Lovley, 2003; Logan *et al*., 2006; Lovley *et al*., 2011). Advances in microbial electrochemical systems have led to a wide range of configurations to desalinate water (Ping *et al*., 2016), harvest energy from soil (Dominguez *et al*., 2014), remove nitrogen from water (Tejedor‐Sanz *et al*. 2016), remediate polluted soils (Rodrigo *et al*., 2014; Rodrigo *et al*., 2016) and synthesize organic compounds from CO_2_ (Patil *et al*., 2015). All of them constitute a plethora of new applications currently the so‐called microbial electrochemical technologies (METs).

Among electroactive bacteria, *G. sulfurreducens* has become a model system for the study of all kinds of METs (Yates *et al*., 2015; Yu *et al*., 2015; Dantas *et al*., 2015; Borjas *et al*., 2015; Estevez‐Canales *et al*., 2015a,b) in order to achieve a deeper understanding of the biological mechanisms involved in direct extracellular electron transfer (DEET; Busalmen *et al*., 2008; Lovley, 2011; Esteve‐Núñez *et al*., 2011; Bond *et al*., 2012; Bonanni *et al*., 2013). In long‐term experiments, with no terminal electron acceptor available apart from the electrode, *G. sulfurreducens* forms a biofilm on the electrode, reaching a thickness of tens of microns (Snider *et al*., 2012; Schrott *et al*., 2014; Stephen *et al*., 2014). The natural formation of an electroactive biofilm, with stable current production, entails prolonged conditioning periods, as long as days or weeks, depending on the architecture of the electrochemical system, and bacterial physiology (Vargas *et al*., 2013; Borjas *et al*., 2015).

With the aim of simplifying this process, recent studies have pointed out the possibility of artificially building electroactive biofilms. Some examples are based on doping the electrode in order to improve the electrical conductivity through the biofilm (Adachi *et al*., 2008; Katuri *et al*., 2011; Liang *et al*., 2011; Nguyen *et al*., 2013). Others have introduced the concept of making an artificial biofilm, where electroactive bacteria are encapsulated into a conductive material to constitute a bioelectrode, as recently demonstrated for *Geobacter* (Srikanth *et al*., 2008) and *Shewanella* (Yu *et al*., 2011; Luckarift *et al*., 2012; Sizemore *et al*., 2013).

Even though biomolecule encapsulation has been studied for several decades (Bjerketorp *et al*., 2006; Nimse *et al*., 2014; Balcão and Vila, 2015; Liu *et al*., 2015), the interest in this methodology for electroactive bacteria such as *Shewanella* (Yu *et al*., 2011; Luckarift *et al*., 2012; Sizemore *et al*., 2013) or *Geobacter* (Srikanth *et al*., 2008) is very recent. Compared with traditional encapsulation, the building up of an artificial electroactive biofilm not only requires preserved bacterial viability but must ensure an efficient electrical contact between the cells and material. There is a wide variety of matrices used for the immobilization of biological species, including agar, pectin, alginate and gelatin (bio‐organic polymers) and silica gels (inorganic polymers; Bjerketorp *et al*., 2006; Homaei *et al*., 2013; Bayat *et al*., 2015).

Organic polymers show good cytocompatibility, allowing solute diffusion and electron exchange (Srikanth *et al*., 2008), but they are prone to swelling and to chemical/biological degradation, making them more interesting for medical applications (Nicodemus and Bryant, 2008; Selimović *et al*., 2012). On the other hand, inorganic polymers, like silica gels, allow both solute diffusion and electron exchange (Ouay *et al*., 2013), but they offer better optical and mechanical properties, making the gel more robust and easier to control than organic polymers (Depagne *et al*., 2012; Wang *et al*., 2015). Silica encapsulation of living bacteria within a conductive support opens the route to ready‐to‐use artificial bioelectrodes, avoiding the long periods typically required for electroactive biofilm formation (Babauta *et al*., 2012). Ready‐to‐use artificial bioelectrodes could be especially useful for biosensing purposes as well as for simplifying the start‐up operation in METs.

To the best of our knowledge, silica gel encapsulation remains unexplored for the whole‐cell immobilization of the model bacteria *G. sulfurreducens*, although its use for immobilizing *Shewanella* in artificial bioelectrodes has been reported (Yu *et al*., 2011; Luckarift *et al*., 2012; Sizemore *et al*., 2013). However, *Shewanella* species are reported to release electron shuttles as the primary mechanism of extracellular electron transfer (Marsili *et al*., 2008; Kotloski and Gralnick, 2013). This may be a drawback in case of serial fed‐batch operation. In contrast, *G. sulfurreducens* establishes DEET via c‐type cytochromes (Busalmen *et al*., 2008) so its performance is not affected when the media is refreshed (Lovley, 2011).

In this work, we report a new approach to construct ready‐to‐use artificial bioelectrodes of *G. sulfurreducens* by means of immobilizing cells in silica gel and carbon felt fibre electrodes.

## Results and discussion

### Survival of *G. sulfurreducens* after encapsulation

**Figure 1:**
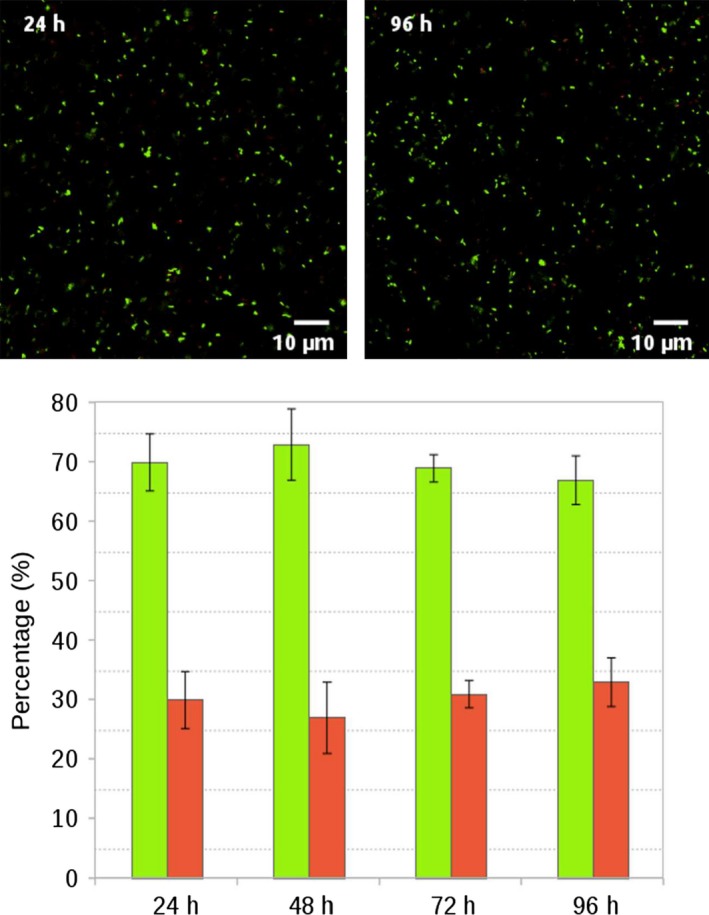
Viability of encapsulated *Geobacter sulfurreducens* within silica gel by Live (green)/Dead (red) staining, up to 96 h.

Cell viability after silica encapsulation was monitored by confocal and multiphoton fluorescence microscopy in the presence of soluble electron donor and acceptor using Live/Dead staining, taking advantage of the suitable optical properties of the silica gel. This kit allows simultaneous observation of living (green) and dead bacteria (red) based on two different fluorescent dyes. Figure 1 shows that the viability after 24 h of encapsulation was reasonably good with the majority of bacteria (ca. 70 ± 5%) surviving the process, in agreement with previous reports on the encapsulation of *E. coli* (Ouay *et al*., 2013) in similar silica matrices. Moreover, 96 h after encapsulation, viability appeared to remain constant, demonstrating that encapsulation in silica gel did not significantly affect *G. sulfurreducens*.

It is also worth noting that cell density did not appear to change over time (Fig. 1). According to the literature, this is due to the fact that in the conditions of encapsulation, the pore size of the silica network (ca. 1–10 nm) is far below the dimensions of bacteria (1–2 μm), the inorganic structure is robust enough to resist cellular deformation forces and silica cannot be biodegraded by most living organisms (Depagne *et al*., 2012; Eleftheriou *et al*., 2013). All these factors prevent in‐gel cell division and proliferation (Wang *et al*., 2015).

### Initial electrochemical response

**Figure 2:**
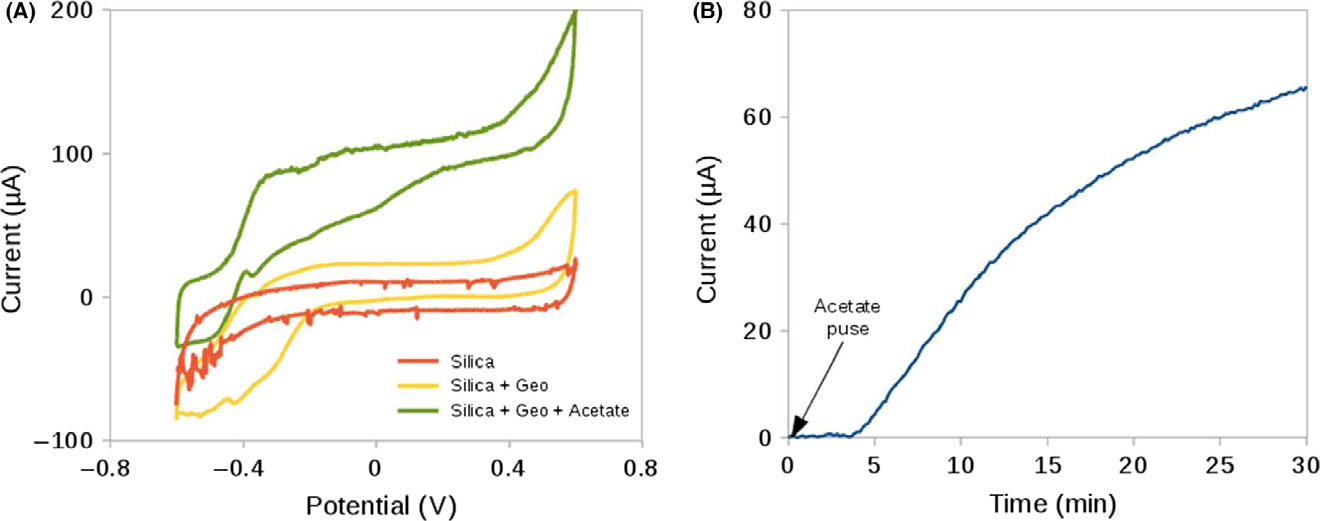
A. Cyclic voltammetries of *Geobacter sulfurreducens* bioelectrodes in the presence of electron donor (green), without electron donor (yellow) and abiotic control with electron donor (red). B. Current production after a pulse of acetate.

Artificial bioelectrodes were assembled by impregnating a hydrophilized carbon felt with a cell suspension mixed with silica precursors. Once the bioelectrodes were assembled, their bioelectrochemical response in the presence of acetate was tested in three‐electrode reactor where the working electrodes were polarized at 0.25 V. Likewise, an abiotic electrode containing silica and acetate, together with a bioelectrode in a free‐acetate reactor, was used as control. After 60 min of polarization at 0.25 V, a cyclic voltammetry analysis was performed in order to explore the catalytic activity of the three conditions. In the presence of acetate, the encapsulated cells displayed a voltammogram with a sigmoidal shape profile, typical of the catalytic activity of an advanced‐stage biofilm of *G. sulfurreducens* (Fig. 2A; Marsili *et al*., 2010; Strycharz *et al*., 2011). These results prove that silica encapsulation does not hinder the electric contact between the bacteria and the electrode and a suitable coupling of acetate metabolism and exocellular electron transfer is still occurring. In contrast, the catalytic activity displayed in the absence of the electron donor (acetate) was lower as expected under a starvation mode physiology.

The diffusion of acetate through the gel was studied in order to confirm that it is not the limiting step in the electroactivity. Accordingly, acetate diffusion from the solution to its conversion to electricity was monitored over time. An assembled bioelectrode was placed in a three‐electrode reactor with no electron donor. First, the cell was polarized at 0.25 V until the complete depletion of intracellular acetate, leading to null current production. Then, a pulse of acetate was added to the reactor to produce a final concentration of 20 mM. As shown in Fig. 2B, the current production was re‐established ca. 5 min after the pulse, revealing that the diffusion of acetate through the silica gel occurred in a short time. Indeed, our results are consistent with other studies conducted on non‐immobilized cells (Bond and Lovley, 2003; Tront *et al*., 2008). This indicates that acetate diffusion from the medium to the encapsulated cells is unlikely to be a limiting step for the characterization of the bioelectrode.

### Electrochemical behaviour of the bioelectrode using lactate and acetate as electron donors

In order to explore the behaviour of the system, the electrochemical activity of the bioelectrode was monitored over time using two different electron donors: acetate and lactate. The artificial bioelectrode was continuously polarized in a three‐electrode reactor for several days. During this time, cyclic voltammetry (CV) and open circuit potential (OCP) measurements were performed every 24 h.

**Figure 3:**
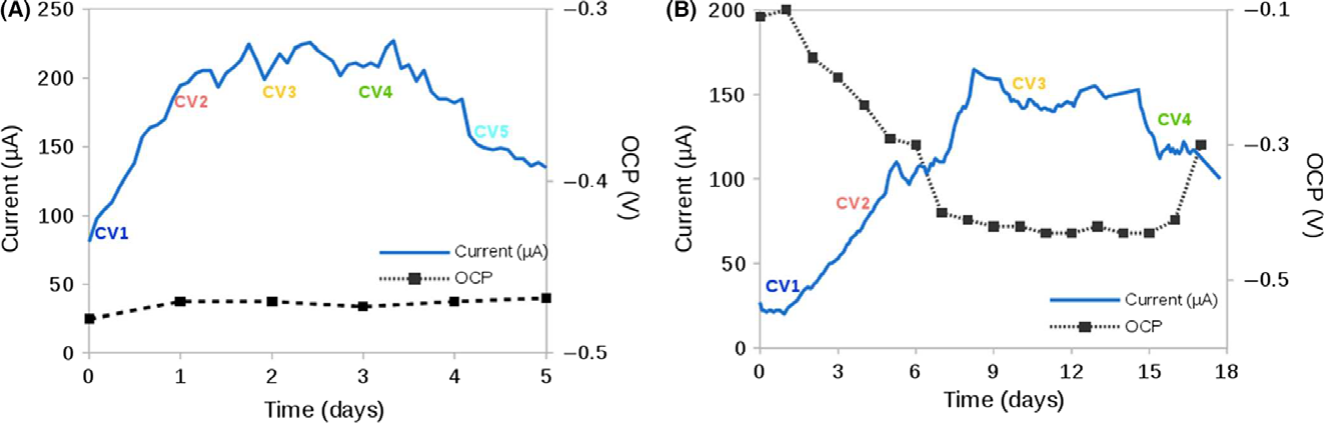
Current production at 0.25 V vs Ag/AgCl and open circuit potential vs time of (A) acetate‐fed artificial bioelectrode and (B) lactate‐fed artificial bioelectrodes.

Acetate is the preferred electron donor for *G. sulfurreducens*, and the end‐product of the acetogenic phase in anaerobic wastewater treatments, so that it is widely used in MET studies (Wang and Ren, 2013; Scott and Yu, 2015). Chronoamperometric results showed a brief initial phase where the current increased, probably reflecting cell adaptation to the system. Maximum current production (ca. 220 μA cm^−3^ bioelectrode) was reached after 1 day of polarization and it remained stable for 3 days. After that period, it started to drop (Fig. 3A). Assuming that cell density remained constant during the experiment, the average oxidation rate of acetate was estimated in ca. 3.5 × 10^−10^ pmol s^−1^ per cell, which is one order of magnitude lower than values for non‐encapsulated cells (Estevez‐Canales *et al*., 2015). This suggests either that encapsulated cells are less active than free cells or that only a fraction of the bacteria are in direct contact with the conductive carbon fibres.

As acetate concentration in the reactor is not limited, biofilm acidification might be a possible cause for the decrease of current production at the final phase. During the reduction of an external electron acceptor, there is a remarkably accumulation of protons that typically limits the performance of MFCs (Franks *et al*., 2008). Opposite, when the terminal electron acceptor is intracellular, like fumarate, those protons are consumed in the process.

The behaviour using lactate was also explored. In *G. sulfurreducens* lactate is metabolized by producing pyruvate and acetate coupled with the reduction of an electrode (Call and Logan, 2011; Speers and Reguera, 2012). Regarding chronoamperometric results, the overall behaviour allows to distinguish again an initial, a steady phase and a drop phase. However, the timescale of these different stages was significantly different from the acetate experiments. The initial phase was considerably longer when lactate was the sole electron donor, and it took ca. 7 days to reach the stable current production (Fig. 3B). Lower steady current (ca. 150 μA cm^−3^ bioelectrode) and oxidation rate (ca. 1 × 10^−10^ pmol s^−1^ per cell) were observed. This is consistent with others studies that attribute this fact to the lactate diversion to anabolic activities rather than to electricity production (Speers and Reguera, 2012).

In both acetate‐ and lactate‐fed bioelectrodes, there was no increase in the turbidity of the media over the time of experiments (data not shown). This clearly indicates that the double entrapment strategy (i.e. cells in a silica gel filling the porosity of a carbon felt) efficiently prevented bacterial leaching from the electrode.

**Figure 4:**
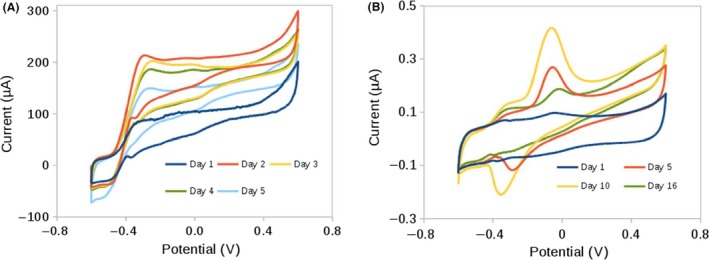
Cyclic voltammetries performed at different times of (A) acetate‐fed artificial bioelectrodes and (B) lactate‐fed artificial bioelectrodes.

In agreement with amperometric results, the voltammograms of the acetate‐fed system exhibit a sigmoidal catalytic wave from the very beginning, with an onset potential near −0.4 V (vs Ag/AgCl). Moreover, they achieved similar limiting current values (ca. 200 μA) as in chronoamperometric assays, as commonly reported for *G. sulfurreducens* biofilms under turnover conditions (Fig. 4A; Katuri *et al*., 2010; Marsili *et al*., 2010). Once the maximum limiting current was reached after 1 day, it remained quite steady for the next 3 days. Again, from this point, limiting current in the CV also decreased, although the OCP (Fig. 3A) remained constant (−0.48 V) during the whole experiment. Similar OCP values have been commonly reported for acetate‐fed biofilms, indicating the sturdiness of our bioelectrode (Babauta *et al*., 2012).

In contrast, lactate‐fed system voltammograms display two well‐defined peak characteristic of the catalytic activity in non‐turnover conditions, when the electron donor is limited in a biofilm (LaBelle and Bond, 2009; Strycharz *et al*., 2011; Fig. 4B).

In agreement with these results, OCP values change towards more negative values until entering in the steady phase, where the potential reaches ca. −0.45 V (vs Ag/AgCl), similar value than the obtained in acetate‐fed system. Meanwhile, higher initial and final values of OCP for lactate‐fed systems could be accounted for a lower concentration of acetate (Fig. 3B).

It is not surprising that in lactate‐fed systems, the electron donor was limited regarding its interaction with the electrode due to metabolic constrains. *G. sulfurreducens* encodes for glycolate oxidase (GO), an enzyme homologous to lactate dehydrogenase in *S. oneidensis* that poorly catalyses the oxidation of lactate (Speers and Reguera, 2012). Moreover, the released pyruvate is described as a poor electron donor for *G. sulfurreducens*, and thus, current contribution in lactate‐fed system is mainly due to its partial oxidation to acetate (Segura *et al*., 2008; Speers and Reguera, 2012). Moreover, in non‐encapsulated mature biofilms, acetate concentrations lower than 3 mM had a great influence on oxidation waves of voltammograms (Marsili *et al*., 2008). The prolonged lag phase required observed in the chroamperometric assay (Fig. 3B) and the voltammetric profile (Fig. 4B) obtained in our work may correspond with a gradual generation of acetate and a low concentration of it.

Metabolic constraints in lactate oxidation coupled with electrode reduction need further study. For instance, they might be partially overcome by adding other carbon sources that displace lactate for anabolic activities (Speers and Reguera, 2012).

### Morphological examination of the artificial bioelectrode

**Figure 5:**
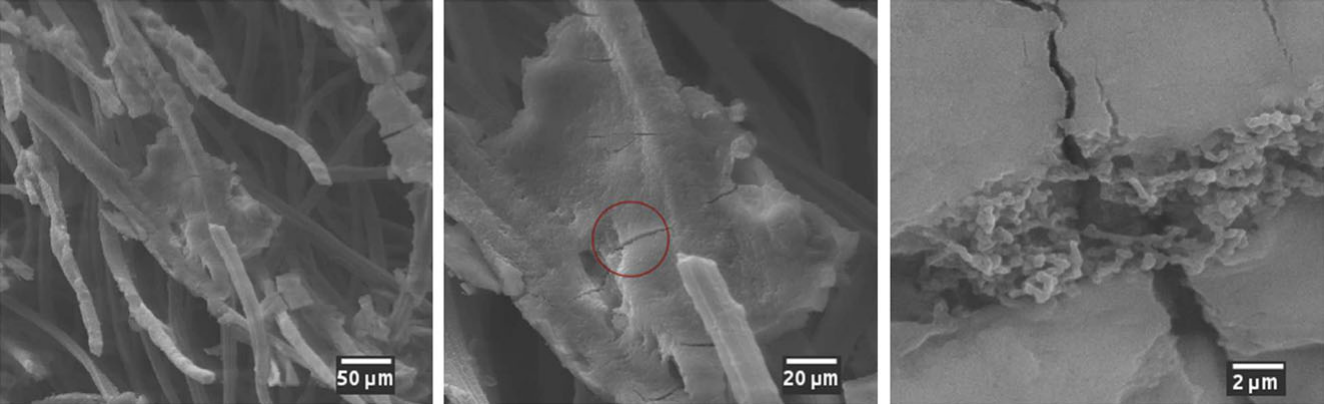
SEM images of the internal morphology of the artificial bioelectrodes.

To further investigate the cell–electrode interface, then the inner structure of a bioelectrode was examined by SEM. The silica material can be easily distinguished from the carbon felt, forming a dense coating on the fibres and sometimes bridging several of them (Fig. 5). At higher magnification, bacteria can be identified in the cracks of the silica grains resulting from the sample drying. Altogether, these images confirm the successful incorporation of silica within the carbon felt and of the bacteria inside the silica network; however, the coverage of the fibres and the distribution of bacteria are heterogeneous. Moreover, the lower acetate oxidation rate estimated in the previous section when compared to reported values for free cells can be partially explained by the low fraction of cells that are able to transfer electrons to the felt.

### Transcriptome analysis

To further understand the cell's response to immobilization, the preliminary transcriptomic response of encapsulated *G. sulfurreducens* was analysed. Transcript abundance of free and encapsulated cells in pure silica gel were analysed. Silica encapsulation resulted in differential expression of 86 genes, 69 overexpressed and 17 underexpressed, considering a *P* value < 0.01. The 30 most differentially expressed genes are listed in Table 1.

**Table 1:** The 30 most strongly overexpressed and underexpressed genes from *Geobacter sulfurreducens* in response to 96 h of silica gel encapsulation compared with a control in standard culture conditions

Gene	Annotation	Fold change	Name
*Overexpressed*			
*GSU0837*	Phosphorelay signal transduction system	8,19	–
*GSU2373*	WHy domain‐containing lipoprotein. Response to desiccation	6,01	–
*GSU2374*	Lytic transglycosylase lipoprotein. Cellular component	5,1	–
*GSU3125*	Mannitol dehydrogenase	4,88	*mdt*
*GSU1142*	Scaffold protein CheW associated with MCPs. Signal transduceractivity	4,74	*cheW34H‐1*
*GSU0663*	Peptidyl‐tRNA hydrolase. Aa translation	4,57	*pth*
*G5U1336*	TerC family integral protein membrane	4,51	–
*GSU0966*	Hypothetical protein	4,36	–
*GSU0832*	Lipoprotein, molecularfuntion lipoprotein	4,31	**–**
*GSU0842*	Phosphorelay signal transduction system	4,3	–
*GSU2863*	DNA‐directed RNA polymerase subunitbeta. Transcription	4,27	*rpoB*
*GSU1141*	Methyl‐accepting sensory transducer. Signal transducer activity	4,19	*mcp34H‐10*
*GSU7603*	3‐oxoacyl‐ACP reductase. Fatty acid biosynthetic process	3,92	*FabG‐2*
*GSU1140*	Methyl‐accepting sensory transducer class 34H. Signal transducer activity	3,83	*mcp34H‐3*
*GSU0151*	Acetylornithine aminotransferase. Arginine biosynthetic process	3,73	*argD*
*GSU0841*	Sigma‐54‐dependenttranscriptional response regulator	3,68	–
*GSU1607*	Serine hydroxymethyltransferase. Glycine biosynthetic process	3,63	*glyA*
*GSU1602*	Malonyl‐CoA carrier protein transacylase. Fatty acid biosynthetic process	3,6	*FabD‐2*
*Underexpressed*			
*GSU3493*	Hypothetical protein	−7,13	–
*GSU0766*	Methyl‐accepting sensory transducer. Signal transducer activity	−5,42	*Mcp64H‐8*
*GSU34W*	Hypothetical protein	−4,87	–
*GSU2529*	Elongation factorG. Translation	−4,62	*FusA‐2*
*GSU0767*	Outer membrane channel protein	−4,4	–
*GSU0593*	Cytochrome b	−4,37	–
*GSU3S60*	Hypothetical protein	−4,01	–
*GSU2470*	Hypothetical protein	−4,01	–
*GSU0768*	Amino acid transmembrane transporter activity	−3,85	–
*GSU2469*	Hypothetical protein	−3,71	–
*GSU1639*	Rrf2 family winged helix–turn–helix transcriptional regulator	−3,2	–
*GSU2590*	Hypothetical protein	−3,2	–

**Figure 6:**
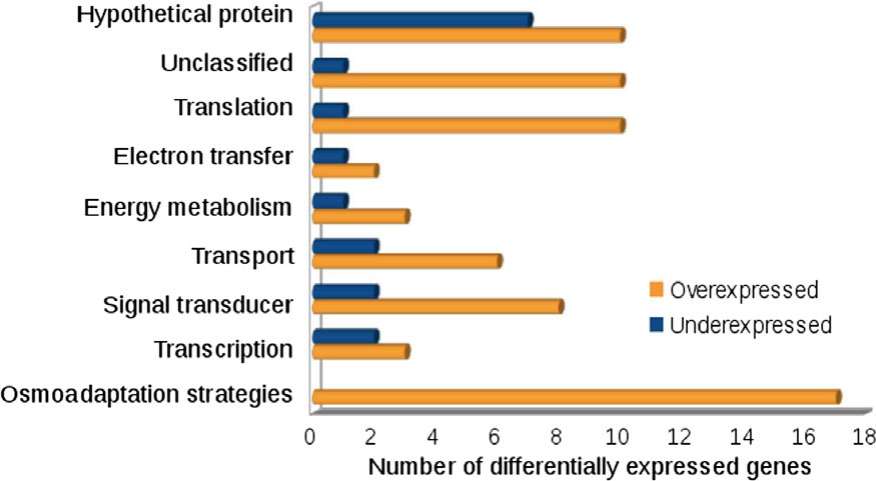
Differential expression of silica‐encapsulated *Geobacter sulfurreducens* according to their annotation function in the genome (NCBI database).

Genes encoding proteins related to osmotic stress are the most strongly overexpressed when *G. sulfurreducens* was encapsulated (Fig. 6). These include genes involved in the synthesis and metabolism of compatible solutes such as amino acids (GSU0151, GSU1607, GSU2371, GSU0153, GSU2874) and sugar alcohol (GSU3125), which were overexpressed between 4.8‐ and threefold. Another bacterial common response to environmental changes, including osmotic stress, is the modification of the membrane composition (Zhang and Rock, 2008). Silica encapsulation of *G. sulfurreducens* triggered the overexpression (between six‐ and 2.9‐fold) of several genes involved in the synthesis and transport of membrane components such as fatty acids (GSU1603, GSU1602, GSU1601), lipoproteins (GSU2373, GSU2374, GSU0832), proteins (GSU1336, GSU2267) and lipopolysaccharides (GSU2085, GSU2490; Fig. 6).

Moreover, bacteria are equipped with a broad number of mechanisms in order to sense information caused by external stimuli into the interior of the cell, and to initiate an according response (Krämer, 2010). Therefore, it is not surprising that several genes involved in signal transduction, translation, transport and transcription exhibited changes in their expression when cells are silica encapsulated, in response to osmotic upshift among other stimuli (Fig. 6).

The transcriptomic results are consistent with previous work that has identified high salinity as the predominant stress for aqueous alkaline silicates precursors of silica gels, like sodium silicate (Dickson *et al*., 2012; Wang *et al*., 2015). Furthermore, similar changes in expression patterns have been recently observed in our research group when *G. sulfurreducens* was cultured in NaCl (Borjas, 2016). These saline‐adapted *G. sulfurreducens* could be used to improve silica gel encapsulation, by avoiding the need for artificial osmoprotectants, and perhaps enhancing the electroactivity of artificial bioelectrodes. Nonetheless, additional analysis of target genes by qRT‐PCR would be required to obtain more conclusive results.

## Concluding remarks

These results show an effective method for the immobilization of *G. sulfurreducens* within silica gel and carbon felt fibres to produce a promising ready‐to‐use artificial bioelectrode. Viability test of silica encapsulation confirmed that the majority of bacteria survived the process and the apparently cell density did not change over time. This double entrapment prevented bacterial release from the electrode, so the system could be electrochemically characterized under suitable mass transport conditions. Whereas *Geobacter*‐based artificial bioelectrodes were tested using acetate and lactate as electron donors, the low oxidation rate and loss of efficiency over time call for further understanding and improvement of the system. Furthermore, the morphology of this artificial bioelectrode was interrogated, revealing a heterogeneous coverage of *G. sulfurreducens* within the fibres and silica gel, so it would be crucial to improve the fraction of silica‐entrapped cells that can contribute to current production. The latter approach is currently under study through the incorporation of conductive elements such as carbon nanotubes or polymer within the silica matrix. Finally, preliminary transcriptomic analysis suggests that osmotic pressure was the predominant stress for silica gel encapsulation of *G. sulfurreducens*.

Our methodology will allow the use of different fibrous conductive materials, silica gel porosity and cell densities, for a wide range of applications (e.g. minimizing the start‐up process of METs, biosensing), avoiding long periods typically required for electroactive biofilm formation. This feature could be useful for conducting basic research as well as for electroanalytical purposes especially for the development of biosensors, as the 3D silica‐electrode encapsulation prevents cell division and the bioelectrode shows a good performance at the short term.

Herein, ready‐to‐use artificial bioelectrodes represent a versatile time‐ and cost‐saving strategy that could be relevant for short‐ and medium‐term experiments in microbial electrochemical systems.

## Experimental procedures

### Bacterial culture

*Geobacter sulfurreducens* (strain DSM 12127; ATCC 51573) was grown at 30°C in freshwater medium containing the following mineral salts (per litre): 2.5 g of NaHCO_3_, 0.25 of NH_4_Cl, 0.06 g of NaH_2_PO_4_H_2_O, 0.1 g of KCl, 0.024 g of C_6_H_5_FeO_7_ (ferric citrate), 10 ml of a vitamin mix and 10 ml of a trace mineral solution (Lovley and Phillips, 1988). Acetate (20 mM) was supplied as the sole carbon source and electron donor, and fumarate (40 mM) as the sole electron acceptor. Anaerobic conditions were achieved by flushing the media with N_2_‐CO_2_ (80:20) to remove oxygen and to keep the pH of the bicarbonate buffer at pH 6.8.

### Bioelectrode construction

First, the carbon felt electrode (1 cm^3^) was treated with an inorganic acid in order to make it more hydrophilic, increasing surface oxidation of the material (Wang *et al*., 2016). Carbon felt (Mersen) was immersed in nitric acid (65%, Sigma‐Aldrich, St. Louis, Missouri, USA) for 48 h. Then, the felt was rinsed with bicarbonate buffer (pH 6.8) and stored in the same solution before use.

Prior to the gel encapsulation, early stationary bacterial cultures were harvested by centrifugation at 8000 rpm for 10 min. These samples were resuspended (OD_600_ = 5) in 90 mM phosphate buffer (pH = 6.8). An anoxic chamber filled with N_2_‐CO_2_ (80:20), (Coy) was used in order to maintain anaerobic conditions, providing an atmosphere of 0–5 parts per million (ppm) using a palladium catalyst and hydrogen gas mix of 5%.

For the encapsulation of *G. sulfurreducens* in silica gel, 0.52 ml of sodium silicate (2 M, Sigma‐Aldrich), 0.1 ml of Ludox HS‐40 (Sigma‐Aldrich) and 1.5 ml of bicarbonate buffer (90 mM) were mixed, deoxygenated and neutralized with 310 μl of HCl (3 M). Subsequently, 2 ml of the concentrated bacterial suspension was added to the mixture, followed by the immersion of the pretreated carbon felt, which lead to a final bacteria concentration of ca. 2 OD_600_ units. Gelation process ends after ca. 20 min, with continuously bubbling with a flux of N_2_‐CO_2_ (80:20) to maintain anoxic conditions inside the bioelectrode. Bioelectrodes were tested immediately after their construction.

### Electrochemical analysis and calculations

All electrochemical assays were performed in a 125 ml three‐electrode reactor. This system consisted of a hermetically sealed glass vessel, where electrodes were assembled. The finished silica–carbon bioelectrode (1 cm^3^) was used as a working electrode, a carbon plate (5 × 2 × 0.5 cm) as counter electrode and an Ag/AgCl 3M reference electrode (BASI). Stainless steel wires were used for the connections of the silica–carbon bioelectrode and they were poked into the carbon felt once the gelation process was concluded. Copper wires used for the connections of the counter electrode were sealed with a conductive epoxy resin (Circuit Works) and isolated with a non‐conductive epoxy resin (Araldit Ceys, Barcelona, Spain).

The system was controlled by a PC‐connected potentiostat (Bio‐Logic Science Instruments, SP‐150). The reactor was filled with 125 ml of 90 mM bicarbonate buffer amended, when specified, with sodium acetate (20 mM) or lactate (20 mM).

All potentials were quoted versus an Ag/AgCl electrode. For chronoamperometric assays, the current was recorded every 10 s and the potential was fixed at 0.25 V. To determine the volumetric current density, it was considered the volume of the carbon felt electrode (1 cm^3^). For cyclic voltammetry, the initial potential was 0 V and the potential window was scanned between −0.6 and 0.6 V at 0.005 V s^−1^. All experiments were performed under a continuous bubbling of N_2_‐CO_2_ (80:20) to maintain an anoxic environment and a pH of 7. All the electrochemical experiments were performed in triplicate using a new bioelectrode for each experiment. Faraday's law (Q = ∫ Idt = n_e_ N_mol_ F) was used for the oxidation rate estimations.

### Viability test

Silica gel‐encapsulated *G. sulfurreducens* was stored under anaerobic conditions using an anoxic chamber (Coy), and viability was tested at 24, 72 and 96 h in the presence of an electron donor and an acceptor. An aliquot of each sample was removed from the bulk of the gel and was fluorescently stained with the LIVE/DEAD Baclight bacterial viability kit (Invitrogen, Thermo Fisher Scientific, Waltham, Massachusetts, USA). For staining mixture, 3 μl of SYTO^®^ 9 and 3 μl of propidium iodide were mixed in 1 ml of bicarbonate buffer (90 mM). The samples of encapsulated bacteria were stained for 1 h at room temperature in the dark under anaerobic conditions. Post‐staining samples were washed twice with bicarbonate buffer, in order to remove the excess of staining, which might cause background noise at the observation. Confocal images were captured using a confocal and multiphoton fluorescence microscope (Leica TCS SP5, Wetzlar, Germany). Images were analysed by the imagej software (Public domain) software for the semiquantitative analysis. Standard deviation was calculated from three biologically independent viability test experiments.

### Scanning Electron Microscopy

A functional bioelectrode was extracted from the reactor, after 72 h of polarization (steady current producing) using acetate as electron donor. Samples were immersed at room temperature for 1 h in cacodylate buffer (0.2 M, pH 7.2) containing 5% glutaraldehyde for the cellular fixation. Samples were rinsed twice in 0.2 M cacodylate buffer, pH 7.2 for 10 min. Samples were then dehydrated with a series of ethanol solutions (25%, 50%, 70%, 90% and 100%) for 10 min at each stage. Finally, ethanol was removed by evaporation at room temperature, before the samples were cut in half and imaged with a scanning electron microscope DSM‐950 (Zeiss, Oberkochen, Germany).

### RNA extraction and transcriptomic analysis

Encapsulated and free *G. sulfurreducens* cells were incubated at 30°C for 96 h in the presence of acetate (20 mM) and fumarate (40 mM) prior to RNA extraction. To harvest RNA, 500 μl of aliquots was scraped (encapsulated sample) or pipetted (free culture sample) into 1.5 ml tubes. Samples were homogenized by the addition of 500 μl of Purezol (BioRad, Hercules, California, USA) and vortexed in the presence of glass beads (1 mm diameter, Sigma‐Aldrich) for 2 min. Samples were then incubated at 65°C for 10 min with occasional mixing. About 100 μl of chloroform (Sigma‐Aldrich) was added into each tube, vortexed for 15 s, placed on ice for 10 min and centrifuged for 15 s at 15,000*xg*. The aqueous phase of each tube (approximately 200 μl) was mixed with 500 μl of 2‐propanol (Sigma‐Aldrich). Then, samples were transfer to RNeasy spin columns (RNeasy kit; Qiagen, Venlo, Netherlands) and centrifuged for 15 s at 8000 × *g*, discarding the eluate. RNA was purified and concentrated in RNase‐free water, following the protocol provided in the RNeasy kit (Qiagen). RNA yield was measured by absorbance at 260 nm (NanoDrop ND‐100; Thermo Fisher Scientific, Waltham, Massachusetts, USA). RNA purity was assessed by examining A260/280 ratio with both samples exceeding a 1.8 value. RNA extracts were sent to FPCM (Campus Cantoblanco, Madrid) to perform the RNA sequencing (RNA‐seq). Bioinformatics analysis was performed by Era7 Bioinformatics (Granada, Spain) and FASTQ files collected after RNA‐seq were analysed using cuffdiff 2.2.1 (Public domain). Annotation of genes was extracted from NCBI database.

## Conflict of interest

The authors declare no conflict of interest.
